# Maternal and perinatal outcomes of low-dose aspirin plus low-molecular-weight heparin therapy on antiphospholipid antibody-positive pregnant women with chronic hypertension

**DOI:** 10.3389/fped.2023.1148547

**Published:** 2023-05-05

**Authors:** Shangqin Long, Liren Zhang, Xiaodong Li, Yongjie He, Xin Wen, Nannan Xu, Xiaoqing Li, Jingmin Wang

**Affiliations:** ^1^ Department of Obstetrics and Gynecology, The First Affiliated Hospital of Dalian Medical University, Dalian, China; ^2^ Department of Obstetrics and Gynecology, Dalian City Third People's Hospital, Dalian, China; ^3^ Department of Urology, Dalian City Third People's Hospital, Dalian, China

**Keywords:** chronic hypertension, low-dose aspirin, low-molecular-weight heparin, antiphospholipid antibody, pregnant women

## Abstract

**Objectives:**

Positive antiphospholipid antibodies (aPLs) and chronic hypertension (CH) in pregnancy are important causes of maternal and neonatal morbidity and mortality. However, there are no relevant studies on the treatment of aPL-positive pregnant women with CH. This study aimed to determine the effect of low-dose aspirin (LDA) plus low-molecular-weight heparin (LMWH) on maternal and perinatal outcomes in persistently aPL-positive pregnant women with CH.

**Methods:**

This study was performed at the First Affiliated Hospital of Dalian Medical University in Liaoning, China, from January 2018 to December 2021. Pregnant women diagnosed CH and persistently positive aPL who had no autoimmune disease such as systemic lupus erythematosus, antiphospholipid syndrome were recruited and divided into control group (LDA and LWMH were not used), LDA group (LDA was used) and LDA plus LMWH group (both LDA and LMWH were used) according to whether they use LDA and/or LMWH. A total of 81 patients were enrolled, including 40 patients in the control group, 19 patients in the LDA group, and 22 patients in the LDA plus LMWH group. The maternal and perinatal outcomes of LDA plus LMWH therapy were analysed.

**Results:**

Compared with control group, the rate of severe preeclampsia in LDA group (65.00% vs. 31.58%, *p* = 0.016) and LDA plus LMWH group (65.00% vs. 36.36%, *p* = 0.030) had a statistically significant reduction. Compared with control group, the rate of fetal loss in LDA group (35.00% vs. 10.53%, *p* = 0.014) and LDA plus LMWH group (35.00% vs. 0.00%, *p* = 0.002) had a statistically significant reduction. Compared with control group, the rate of live birth in LDA group (65.00% vs. 89.74%, *p* = 0.048) and LDA plus LMWH group (65.00% vs. 100.00%, *p* = 0.002) had a statistically significant increased. Compared withcontrol group, the incidence of early-onset preeclampsia (47.50% vs. 36.84%, *p* = 0.008) and early-onset severe preeclampsia (47.50% vs. 13.64%, *p* = 0.001) in the LDA plus LMWH group decreased and were statistically different. Furthermore, we also found that LDA or LDA plus LMWH hadn't increase the rate of blood loss and placental abruption.

**Conclusion:**

Both LDA and LDA combined with LMWH could decrease the incidence of severe preeclampsia, decrease the rate of foetal loss, increase the rate of live birth. However, LDA plus LWMH could reduce and delay the onset of severe preeclampsia, prolong the gestational age and increase the rate of full-term delivery, improve the maternal and perinatal outcomes.

## 1. Introduction

Hypertensive disorders are the most common medical complication of pregnancy and represent one of the major global causes of maternal and perinatal morbidity and mortality (1, 2). Recently, the principal international boards of obstetrics and gynaecology reached a consensus on the diagnostic criteria, wherein HDP is considered a group of diseases that coexist with pregnancy and elevated blood pressure, including gestational hypertension (pregnancy-induced hypertension, chronic hypertension [CH] complicating pregnancy), preeclampsia (PE; preeclampsia *de novo*, superimposed preeclampsia on CH) and eclampsia (3, 4). CH refers to hypertension (> 140/90 mmHg) before pregnancy or before 20 weeks of gestation and persisting>12 weeks after delivery (5). According to the existing literature, CH complicates 1% to 2% of pregnancies and constitutes the highest risk factor, among maternal characteristics and medical history, for the development of PE. A recent study showed that severe PE occurs in about 20% of women with CH, and the risk of preterm delivery is 5 to 6 times higher in women with CH superimposed with PE compared with those without PE (5).

aPLs are a group of autoantibodies against a variety of negatively charged phospholipids, including lupus anticoagulant (LA), anticardiolipin antibodies (aCL), anti-β2 glycoprotein-I antibodies (anti-β2 GPI), anti-phosphatidylserine (aPS), anti-phosphatidylinositol (API), anti-phosphatidylethanolamine (APE) etc. (6, 7), A recent study proposed the inclusion of LA, aCL and anti-β2 GPI antibodies in the laboratory diagnosis of APS (8). Most studies have shown that patients with PE have a high rate of aPL positivity than the general population (9). Among them, patients with aPL positivity have a higher risk of early-onset severe PE (9). Duckitt K et al. found that women with aPLs have an almost ten-fold increased risk of PE (10). A recent study reported that women with these antibodies are at higher risk of recurrent miscarriage, stillbirth, and other pregnancy-associated morbidities, including PE, a pregnancy disorder of maternal high blood pressure and systemic endothelial activation (11). The current guidelines recommend the use of LDA to prevent PE in pregnant women with CH in the first trimester (12). However, aPL-positive pregnant women are treated with a combination of LMWH and LDA, with a success rate of approximately 75% (13). As we all known, CH and APS are both vascular disorders and renal involvement of antiphospholipid antibodies. Malignant hypertension is a hallmark of APS-nephropathy. Several studies have elaborated the reasons why chronic hypertension is associated with aPL (14). Some studies demonstrated that circulating levels of aPL in pregnant women with CH are greater compared to normotensive women (15). In addition, some researchers have listed CH as a clinical feature associated with the presence of aPLs (16). With the improvement and standardization of technologies, aPLs are being identified in an increasing number of women with CH. However, there are no relevant studies regarding the treatment of aPL positivity and CH in pregnancy.

Moreover, there are limited studies and no clear guidelines for the prevention of complications among aPL-positive pregnant women with CH. Therefore, the present study mainly explored the effect of LDA combined with LMWH on pregnancy outcomes in aPL-positive women with CH.

## 2. Material and methods

### 2.1. Study cohort

**Figure 1:**
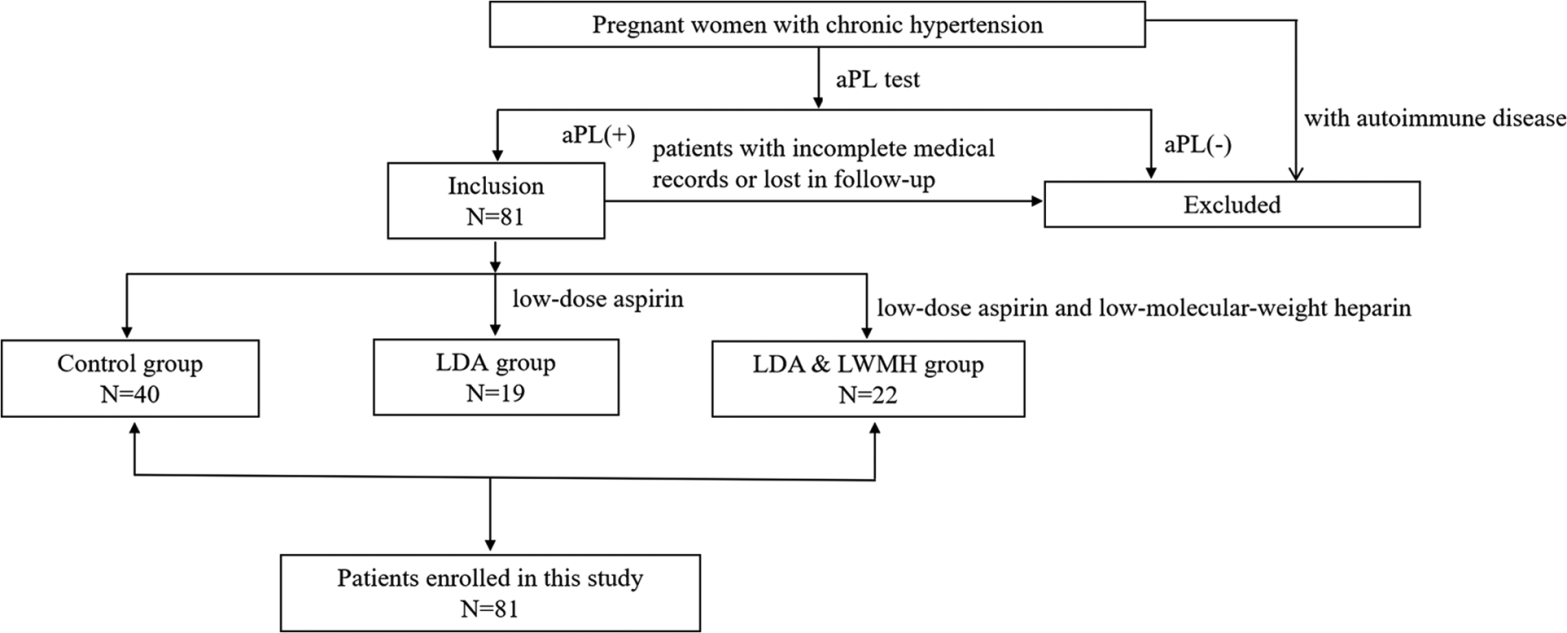
Patient enrolment.

This study included persistently aPL-positive pregnant women with CH who had no autoimmune disease such as systemic lupus erythematosus, antiphospholipid syndrome, myasthenia gravis or pernicious anaemia and delivered in the First Affiliated Hospital of Dalian Medical University in Liaoning, China, from January 2018 to December 2021. The data collection began after obtaining approval from the Ethics Committee of The First Affiliated Hospital of Dalian Medical University. Pregnant women with CH who were positive for aPLs (LA, aCL, and/or anti-β_2_-GPI) were included, while patients with incomplete medical records and those who were lost to follow-up were excluded from this study. If a woman had more than one pregnancy, we considered only the first index pregnancy for them. The flowchart of patient enrolment is shown in Figure 1.

### 2.2. Data collection

Data were collected from the medical records of the patients. Data on the general maternal characteristics (including age, BMI, blood pressure and pre-pregnancy complications, such as chronic kidney disease, diabetes mellitus, hypothyroidism etc.) were recorded to assess baseline uniformity. The primary outcomes of this study included maternal outcomes (PE, early-onset PE, severe PE, early-onset severe PE, eclampsia, thrombocytopenia, HELLP syndrome, oligoamnios, placental abruption, premature rupture of membranes, heart failure and postpartum hemorrhage, among other outcomes), and perinatal outcomes (gestational age, baby birth weight, foetal loss, prematurity, caesarean delivery and foetal growth restriction [FGR], among other outcomes). At discharge, clinical diagnosis was determined according to Table 1.

**Table 1:** Inclusion diagnosis and criteria at discharge.

Inclusion diagnosis	Clinical criteria
CH in pregnancy (17)	CH diagnosed before pregnancy or before 20 weeks' gestation, or first diagnosed after 20 weeks' gestation and lasts 12 weeks postpartum
PE (18)	Which was based on the new onset of proteinuria (dipstick test> + 2, 300 mg or more per 24 h urine collection or protein/creatinine ratio of 0.3 or more), maternal organ dysfunction (renal insufficiency, liver involvement, pulmonary oedema, neurological conditions, thrombocytopenia, etc.)
Early-onset PE (11)	Severe PE was diagnosed and delivery at <34 + 0 weeks of gestation
Severe PE (18)	Systolic blood pressure ≥180 mmHg, and/or Diastolic blood pressure ≥110 mmHg and proteinuria (≥0.3 g/24 h)
Early-onset severe PE (11)	Severe PE was diagnosed and delivery at <34 + 0 weeks of gestation
Eclampsia (18)	Generalized convulsions in pregnancy not caused by epilepsy
FGR (19)	Ultrasound estimated foetal weight at inclusion or birth weight at delivery <10th centile

### 2.3. Statistical analysis

Data were analysed using IBM SPSS Statistics® 26. The unpaired t-test was used for quantitative data to evaluate differences between two groups with normal distribution and the relation between two parameters. The *x^2^* test was used to evaluate qualitative data. The Mann-Whitney *U* test was used for variables with an abnormal distribution.

## 3. Results

### 3.1. Basic clinical data

Between January 2018 and December 2021, a total of 524 pregnant women were diagnosed with chronic hypertension in our hospital, and 34 patients had autoimmune diseases, we ruled them out. A total of 490 patients were enrolled. In these 490 patients, only 184 patients were screened for aPL, the examination rate was 37.55%. In these 184 patients, 81 women were aPL positive with an interval of more than 12 weeks. the rate of aPL positive in pregnant patients with chronic hypertension was 44.02%. In these 81 patients enrolled, 19 were treated with low-dose aspirin and were included in the LDA group, 22 were treated with low-dose aspirin and low-molecular-weight heparin and were included in the LDA plus LMWH group, and 40 women who were not treated with aspirin or LMWH were allocated to the control group. The basic clinical date between the three group, including age, BMI, blood pressure stage and pre-pregnancy complications, were not significantly different (Table 2). Further, we compared the maternal and perinatal outcomes in the three groups.

**Table 2:** Basic clinical data of the three groups.

Characteristics	Control group	LDA group	LDA + LMWH group	*P*
Cases	40	19	22	
Age (years), (¯*x* *±* s)	33.83 ± 5.70	34.25 ± 4.99	31.38 ± 3.35	0.187
BMI(kg/m^2^), (¯*x* *±* s)	27.21 *±* 4.91	26.67 *±* 5.56	27.43 *±* 4.39	0.114
No. of previous pregnancy failures				
1 to 2 times before 10 weeks	9	3	4	0.813
≥3 times before 10 weeks	0	0	1	0.257
Pregnancy failures after 10 weeks	1	2	4	0.104
Hypertension grade, *n* (%)				
Grade 1	2 (5.00%)	1 (5.26%)	1 (4.55%)	0.994
Grade 2	14 (35.00%)	6 (31.58%)	9 (40.91%)	0.815
Grade 3	24 (60.00%)	12 (63.16%)	12 (54.55%)	0.847
Complications				
CKD, *n* (%)	6 (15.00%)	5 (26.32%)	4 (18.18%)	0.578
Diabetes mellitus, *n* (%)	3 (7.50%)	5 (26.32%)	4 (18.18%)	0.143
Gestational diabetes mellitus, *n* (%)	9 (22.50%)	5 (26.32%)	3 (13.64%)	0.578
Obesity, *n* (%)	15 (37.50%)	7 (36.84%)	10 (45.46%)	0.799
Hypothyroidism, *n* (%)	6 (15.00%)	3 (15.79%)	4 (18.18%)	0.947

BMI, body mass index; CKD, chronic kidney disease; hypertension grade 1, hypertension was defined as a systolic blood pressure of 140–159 mmHg or diastolic blood pressure of 90–99 mmHg, hypertension stage 2: a systolic blood pressure of 160–179 mmHg or diastolic blood pressure of 100–109 mmHg, hypertension stage 3: a systolic blood pressure ≥180 mmHg and/or diastolic blood pressure ≥110 mmHg, based on at least two measurements taken within15 min apart.

### 3.2. Maternal and perinatal outcomes in the three groups

In this study, we found that in aPL-positive pregnant women with CH, both LDA or LDA in combination with LWMH can reduce the rate of severe preeclampsia, reduce fetal loss rates, increase live birth rates and average birth weight. Furthermore, the use of LDA combined with LMWH can reduce the incidence of early-onset preeclampsia and early-onset severe preeclampsia. The use of LDA plus LMWH can increase the gestational age and full-term delivery rates. Suggesting that in patients with aPL-positive pregnancy with chronic hypertension, LDA alone is not enough for avoiding the early-onset preeclampsia and early-onset severe preeclampsia. It is recommended that aPL should be screened in pregnant patients with chronic hypertension, once abnormal aPL results are found, LDA and LMWH should be added promptly.

In maternal outcomes, we found that compared with the control group, the rate of severe preeclampsia in LDA group (65.00% vs. 31.58%, *p* = 0.016) and LDA plus LMWH group (65.00% vs. 36.36%, *p* = 0.030) had a statistically significant reduction. However, when compared with the LDA group, there were no significant differences in the incidence of severe preeclampsia in LDA plus LMWH group. compared with the control group, the incidence of early-onset preeclampsia (47.50% vs. 36.84%, *p* = 0.008) and early-onset severe preeclampsia (47.50% vs. 13.64%, *p* = 0.001) in pregnant women in the LDA plus LMWH group decreased and were statistically different. However, when compared with the control group, the incidence of early-onset preeclampsia and early-onset severe preeclampsia in the LDA group had no statistically significant difference. Moreover, the incidence of thrombocytopenia, HELLP syndrome, oligoamnios, placental abruption, rupture of membranes, heart failure and postpartum haemorrhage were not statistically significant in LDA group and LDA plus LMWH group when compared with control group (Table 3).

**Table 3:** Maternal and perinatal outcomes of patients in the three groups.

Outcomes	Con	LDA	LDA & LMWH	*P*
Cases	40	19	22	
Maternal outcomes				
Preeclampsia, *n* (%)	31 (77.50%)	12 (63.16%)	11 (50.00%)	0.083
Early-onset PE, *n* (%)	19 (47.50%)	7 (36.84%)	3*(13.64%)	0.029
Severe preeclampsia, *n* (%)	26 (65.00%)	6* (31.58%)	6* (36.36%)	0.020
Early-onset severe PE, *n* (%)	16 (40.00%)	4 (21.05%)	2* (9.09%)	0.026
Thrombocytopenia, *n* (%)	1 (2.50%)	0 (0.00%)	1 (4.55%)	0.646
HELLP syndrome, *n* (%)	9 (22.50%)	1 (4.35%)	1 (4.55%)	0.069
Oligoamnios, *n* (%)	4 (10.00%)	1 (5.26%)	3 (13.64%)	0.669
Placental abruption, *n* (%)	1 (2.50%)	1 (5.26%)	1 (4.55%)	0.845
Rupture of membranes, *n* (%)	2 (5.00%)	1 (5.26%)	1 (4.55%)	0.994
Heart failure, *n* (%)	3 (7.50%)	1 (4.35%)	0 (0.00%)	0.426
Postpartum haemorrhage, *n* (%)	1 (2.50%)	0 (0.00%)	1 (4.55%)	0.646
Perinatal outcomes				
Gestational age (weeks), (*x̄* *±* s)	31.00 ± 7.14	34.92 ± 4.24	35.78 ± 3.67*	0.005
Average birth weight (kg), (*x̄* *±* s)	1.65 ± 0.18	2.35 ± 0.27*	2.46 ± 0.21*	0.012
Foetal loss, *n* (%)	14 (35.00%)	2* (10.53%)	0* (0.00%)	0.002
Live birth, *n* (%)	26 (65.00%)	17* (89.47%)	22* (100.00%)	0.001
Preterm birth, *n* (%)	18 (45.00%)	9 (47.37%)	7 (31.84%)	0.520
Before 34 weeks, *n* (%)	10 (25.00%)	5 (26.32%)	6 (27.27%)	0.980
34–37 weeks, *n* (%)	8 (20.00%)	4 (21.05%)	1 (4.55%)	0.226
Full-term delivery, *n* (%)	8 (20.00%)	8 (42.11%)	15* (68.18%)	0.000
Caesarean delivery, *n* (%)	21 (52.50%)	16^a^ (84.21%)	19* (86.36%)	0.006
FGR, *n* (%)	11 (27.50%)	6 (31.58%)	8 (36.36%)	0.768
Blood loss in the first 24 h after delivery, *n* (%)				
Antepartum Hb, (*x̄* *±* s)	126.70 ± 13.97	122.95 ± 15.75	123.64 ± 16.00	0.525
Postpartum Hb, (*x̄* *±* s)	115.05 ± 14.88	108.26 ± 15.30	113.18 ± 16.55	0.177
*δ* Hb, (*x̄* *±* s)	11.65 ± 8.75	14.68 ± 10.57	10.46 ± 8.84	0.431

FGR: foetal growth restriction.

* Compared with the control group, the results were statistically significant.

In perinatal outcomes, we found that compared with the control group, the rate of fetal loss in LDA group (35.00% vs. 10.53%, *p* = 0.014) and LDA plus LMWH group (35.00% vs. 0.00%, *p* = 0.002) had a statistically significant reduction, However, when compared with the LDA group, there were no significant differences in the incidence of the rate of fetal loss. Compared with control group, the rate of live birth in LDA group (65.00% vs. 89.74%, *p* = 0.048) and LDA plus LMWH group (65.00% vs. 100.00%, *p* = 0.002) had a statistically significant increased. Compared with control group, the average birth weight in LDA group (1.65 ± 0.18 vs. 2.35 ± 0.27, *P* = 0.016) and LDA plus LMWH group (1.65 ± 0.18 vs. 2.46 ± 0.21, *P* = 0.007) had a statistically significant increased. However, when compared with the LDA group, there were no significant differences in the rate of fetal loss and the rate of live birth. Compared with control group, the gestational age (31.00 ± 7.14 vs. 35.78 ± 3.67, *P* = 0.024) and the rate of full-term delivery (20.00% vs. 68.18%, *p* = 0.001) in pregnant women in the LDA plus LMWH group decreased and were statistically different. However, when compared with the control group, the incidence of gestational age and the rate of full-term delivery in the LDA group had no statistically significant difference. However, there were no significant differences were observed in the incidence of preterm birth, caesarean delivery and FGR between the three groups (Table 3).

Furthermore, we found that there had no significant difference in blood loss and placental abruption between the three groups.

## 4. Discussion

In the present study, we assessed the maternal and perinatal outcomes in 81 patients aiming to determine the effect of LDA and LDA combined with LMWH in aPL-positive pregnant women with CH. We observed that both LDA and LDA combined with LMWH could reduce the rate of foetal loss and increase the average birth weight compared to control group. Furthermore, LDA combined with LMWH could reduce the rates of early-onset PE and early-onset severe PE in aPL-positive pregnant women with CH and improve maternal and perinatal outcomes.

It is known that the presence of aPLs is associated with a hypercoagulable state, referring to an abnormally increased tendency toward blood clotting (20). Maternal aPL positivity has been associated with the development of placental insufficiency, characterised by abnormal uteroplacental vascular function, resulting in serious pregnancy complications, including PE and FGR, which are major causes of maternal and foetal mortality and morbidity (21). Aspirin can suppress the production of thromboxane, leading to an inhibition of platelet aggregation, thereby producing an antithrombotic effect due to the inactivation of cyclooxygenase (COX)-1 and COX-2 enzymes (22, 23). LMWH possesses anti-inflammatory and antithrombotic properties (24). A recent study proved that LMWH prevents metabolic and immunological disorders causing placental inflammation, thereby reducing pregnancy complications (25). Initially, LDA and LMWH were considered an effective and safe treatment of aPL- positive women (26).

In this study, we found that there had no significant differences in the rate of preeclampsia in both LDA group and LDA plus LMWH group compared with control group. Our study also observed that both LDA and LDA combined with LMWH could reduce the rate of severe preeclampsia, furthermore, the use of LDA combined with LMWH can reduce the incidence of early-onset preeclampsia and early-onset severe preeclampsia in aPL-positive pregnant women with CH, however, there had no significant differences in the rate of early-onset preeclampsia and early-onset severe preeclampsia in LDA group when compared with control group. Therefore, we concluded that LDA could reduce the incidence of severe preeclampsia to a certain extent, however, LDA plus LMWH can delay the onset of severe preeclampsia and improve the perinatal outcomes in aPL-positive pregnant women with CH. Similarly, other studies reported that LAD combined with LMWH improved maternal outcomes in pregnant women with CH (27, 28). Poon LC et al. reported that LDA did not reduce the incidence of preterm PE in women with CH (28). similarly, Lu C et al. reported that aspirin therapy alone did not demonstrate a significant statistical difference in placenta-mediated pregnancy complications compared to placebo among women with aPL positivity (27). The study by other researchers also showed that the combination of LDA and LMWH can reduce the occurrence of preeclampsia, which is similar to this study (29, 30). Nevertheless, for the first time, the present study observed that LDA combined with LMWH could reduce the rate of early-onset preeclampsia and early-onset severe preeclampsia in aPL-positive pregnant women with CH.

In this study, we observed that both LDA and LDA combined with LMWH could reduce the rate of foetal loss and increase the rate of live birth and the foetal average birth weight compared to control group. However, there was no significant difference between these perinatal outcomes with the use of LDA plus LMWH vs. aspirin monotherapy. Therefore, we concluded that LDA or LDA plus LMWH could improve the perinatal outcomes to a certain extent. Similarly, previous studies have reported that, compared with the placebo group, LDA combined with LMWH reduced the rate of miscarriage, increased neonatal weight and improved perinatal outcomes in aPL-positive pregnant women (31–33). Other studies reported that LAD combined with LMWH improved perinatal outcomes in pregnant women with CH (34). Shi T et al. conducted a study on the prevention of maternal outcomes and found that LDA combined with LMWH could reduce the rate of foetal loss and improve the live birth rate in aPL-positive pregnant women (32). On the other hand, in aPL-positive pregnant women with CH, we found that LDA combined with LMWH could reduce the rate of foetal loss, prolong the gestational age, increase the average birth weight and the rate of full-term delivery, however, LDA alone had no statistically significant effect ongestational age and foetal average birth weight. This finding is consistent with those reported in a recent systematic review (13).

Finally, this study showed that treatment with LDA or LDA combined with LMWH does not increase the risk of postpartum haemorrhage and placental abruption, similar to that reported in many previous studies (35, 36).

Although this study is the first-ever synopsis to highlight the roles of both LDA and LDA combined with LMWH in improving maternal and perinatal outcomes, certain limitations should be noted. Due to the limited sample size, more large-scale prospective studies are required to confirm the conclusions of our study. In addition, we only conducted experimental verification at the level of clinical expression. In the future, more in-depth fundamental investigations will be required to evaluate and confirm the precise mechanism of LDA and LDA combined with LMWH on aPL-positive pregnant women with CH.

## 5. Conclusion

We observed that both LDA and LDA combined with LMWH could decrease the incidence of severe preeclampsia, decrease the rate of foetal loss, increase the rate of live birth and increase the average birth weight. However, LDA plus LWMH could reduce and delay the onset of severe preeclampsia, prolong the gestational age and increase the rate of full-term delivery, improve the maternal and perinatal outcomes.

## Data Availability

The original contributions presented in the study are included in the article, further inquiries can be directed to the corresponding author.
